# Semaglutide in Adolescents Living With Obesity: A Real‐World Data Study Exploring Predictors of Treatment Response

**DOI:** 10.1111/dom.70926

**Published:** 2026-06-03

**Authors:** Valeria Cimador, Sophie Robertson, Christine Desmond, Jessica Dodgson, Elizabeth van Boxel, Nikki L. Davis, Rebecca J. Moon

**Affiliations:** ^1^ Regional Centre for Paediatric Endocrinology, Southampton Children's Hospital University Hospital Southampton NHS Foundation Trust Southampton UK; ^2^ Residency School of Pediatrics University of Bologna Bologna Italy; ^3^ Department of Paediatrics Portsmouth Hospitals University NHS Trust Portsmouth UK; ^4^ MRC Lifecourse Epidemiology Centre University of Southampton Southampton UK; ^5^ NIHR Southampton Biomedical Research Centre University of Southampton and University Hospital Southampton NHS Foundation Trust Southampton UK

**Keywords:** children and adolescents, glucagon‐like peptide 1 receptor agonist, obesity, semaglutide, weight loss

## 1 Introduction

Glucagon‐like peptide‐1 receptor agonists (GLP‐1RA) are promising pharmacologic interventions for obesity. Semaglutide improved body mass index (BMI) and cardiometabolic risk factors in adolescents in a formal trial [1]. Drug effectiveness often differs in routine care to trial settings, particularly when used in more heterogeneous populations, yet real‐world data from GLP‐1RA use in adolescents remains sparse [2, 3, 4]. We evaluated weight and BMI changes with semaglutide use in adolescents and explored patient factors associated with treatment response.

## 2 Methods

We analysed routinely collected clinical data for patients < 18 years treated with semaglutide at two United Kingdom hospitals: University Hospital Southampton NHS Foundation Trust and Portsmouth Hospitals University NHS Trust. Height, weight, medical diagnoses and prescribed medications were recorded at baseline (prior to commencing semaglutide) and at each follow‐up appointment. Disordered eating behaviours (self/parent‐reported hyperphagia and emotional overeating) were noted from clinical documentation. Height, weight and BMI *z*‐scores (BMIz) for age and sex were generated using the British 1990 reference data. All data available up to 11 October 2025 were included.

Due to supply issues in 2023–2024, some patients discontinued semaglutide and restarted once supply was re‐established. For patients restarting semaglutide after ≥ 4 weeks, this was considered a second treatment episode.

### 2.1 Statistical Analysis

To facilitate comparison between patients with varying follow‐up lengths, windows of 120–240 days (“6 months”) and 300–420 days (“12 months”) were used, and adjusted weight and BMI changes calculated for treatment duration (absolute change divided by the number of treatment days, multiplied by 180 and 360, respectively). The association between patient characteristics and change in BMIz (ΔBMIz) (standardized using a Fisher‐Yates transformation) was explored using linear regression. Factors with *p* < 0.1 in univariate analysis were included in a multivariate model to determine independent association.

## 3 Results

220 treatment episodes for 197 patients were included. Median age at semaglutide start was 15.1 years (IQR 13.6, 16.7) and 46% male. Baseline mean (SD) weight and BMIz were 113.0 kg (27.6) and 3.55 (0.58), respectively (Table S1). Confirmed/suspected autism (45%) and mental health disorders (20%) were common. Other diagnoses are shown in Table S2.

Data were available for 161 and 85 patients at 6 and 12 months, respectively Figure (S1). Weight and BMI change metrics are shown in Table 1, with comparison to that reported in the STEP‐TEENS trial [1]. Marked variability was found, for example weight change ranged from −30.8 kg to +13.2 kg at 6 months (Figure S2).

**TABLE 1:** Weight and BMI change after 6 and 12 months of semaglutide treatment in this real‐world data set and shown in comparison to the findings of the STEP‐TEENS intervention trial.

	After 6 months of treatment		After 12 months of treatment		68 weeks in the STEP‐TEENS trial^a^	
	*n*		*n*		*n*	
Weight						
Weight loss (kg), mean (SD)	161	6.4 (7.3)	85	10.0 (11.5)	134	15.3
Weight loss relative to starting weight (%), median (IQR)	161	5.1 (1.0, 8.8)	85	8.9 (0.7, 14.1)	134	14.7^‡^
≥ 5% weight loss, *n* (%)	161	82 (50.9)	85	55 (64.7)	Not available	
≥ 10% weight loss, *n* (%)	161	34 (21.1)	85	38 (44.7)	131	81 (61.8)
≥ 15% weight loss, *n* (%)	161	14 (8.7)	85	20 (23.5)	131	49 (37.4)
BMI						
BMI reduction (kg/m^2^), mean (SD)	160	2.6 (2.5)	82	4.3 (3.8)	134	−5.8
BMI reduction relative to starting BMI (%), mean (SD)	160	6.6 (6.5)	82	10.5 (8.9)	134	16.1
BMI *z*‐score reduction, median (IQR)	160	0.21 (0.10, 0.42)	82	0.37 (0.18, 0.66)	134	1.1^b^
Semaglutide dose, *n* (%) 0.25 mg/week 0.5 mg/week 1.0 mg/week 1.7 mg/week 2 mg/week 2.4 mg/week	159	0 9 (5.7) 129 (81.1) 14 (8.8) 3 (1.9) 4 (2.5)	84	2 (2.4) 9 (10.7) 49 (58.3) 13 (15.5) 1 (1.2) 10 (11.9)	Of the 102 that completed 68 weeks of treatment, 86.7% were taking 2.4 mg, 5.0% 1.7‐ < 2.4 mg, 7.6% > 0.0–1.7 mg	

^a^ Data for the STEP‐TEENS trial have been taken from the published manuscript of Weghuber et al. [1]. Only mean values were published.

^b^ Mean values.

Most patients were prescribed submaximal doses (Table 1). Changes in weight and BMIz were numerically greater in patients prescribed ≤ 1 mg/week compared with higher doses, but these differences were not statistically significant (Table S3).

In univariate analysis, baseline BMIz and weight, type 2 diabetes (T2DM) and self‐reported emotional eating behaviours were positively associated with ΔBMIz at 6 months, whereas type 1 diabetes and multiple pituitary hormone deficits were negatively associated. In multivariate analysis, only the association with T2DM remained significant (Table 2). At 12 months of treatment, baseline BMIz and weight, emotional overeating and prescription of melatonin were positively associated with ΔBMIz, whereas female sex was negatively associated. Only female sex remained significant in multivariate analysis (Table 2).

**TABLE 2:** Associations between patient characteristics and change in BMI *z*‐score during 6 and 12 months of semaglutide treatment.

	6 months				12 months			
	Univariate		Multivariate		Univariate		Multivariate	
	Beta (SD)^a^	*p*	Beta (SD)^a^	*p*	Beta (SD)^a^	*p*	Beta (SD)^a^	*p*
Age (years)	0.02 (−0.06, 0.10)	0.61			−0.01 (−0.12, 0.10)	0.87		
Female sex	−0.21 (−0.52, 0.09)	0.17			−0.47 (−0.89, 0.06)	0.03	−0.44 (−0.88, 0.02)	0.04
Baseline BMI *z*‐score	0.32 (0.05, 0.58)	0.02	0.16 (−0.27, 0.60)	0.46	0.40 (0.04, 0.76)	0.03	0.36 (−0.19, 0.90)	0.20
Baseline weight (kg)	0.01 (0.00, 0.01)	0.006	0.00 (−0.00, 0.01)	0.36	0.01 (0.00, 0.02)	0.02	0.00 (−0.01, 0.01)	0.92
Index of multiple deprivation^b^	−0.04 (−0.09, 0.02)	0.17			−0.02 (−0.09, 0.06)	0.68		
Comorbidities								
Type 1 diabetes mellitus	−0.89 (−1.68, −0.10)	0.03	−0.59 (−1.41, 0.22)	0.15	−0.53 (−1.66, 0.59)	0.35		
Type 2 diabetes mellitus	0.44 (−0.05, 0.93)	0.08	0.53 (0.02, 1.04)	0.04	0.13 (−0.85, 1.12)	0.79		
Prediabetes	0.19 (−0.19, 0.57)	0.32			0.19 (−0.32, 0.70)	0.46		
Insulin resistance	−0.06 (−0.37, 0.25)	0.71			0.06 (−0.37, 0.50)	0.77		
Dysglycaemia^c^	0.26 (−0.22, 0.75)	0.28			0.45 (−0.30, 1.21)	0.23		
Hypertension	0.40 (−0.08, 0.87)	0.10			0.36 (−0.31, 1.03)	0.29		
Metabolic dysfunction‐associated steatotic liver disease	0.25 (−0.05, 0.55)	0.11			0.16 (−0.26, 0.59)	0.44		
Autistic spectrum disorder^d^	0.01 (−0.30, 0.31)	0.97			−0.06 (−0.49, 0.37)	0.79		
Attention deficit hyperactivity disorder^d^	0.22 (−0.13, 0.57)	0.22			0.02 (−0.46, 0.50)	0.92		
Hyperphagia	0.13 (−0.28, 0.53)	0.54			0.33 (−0.19, 0.85)	0.21		
Emotional overeating	0.46 (0.04, 0.89)	0.03	0.33 (−0.09, 0.74)	0.12	0.51 (−0.05, 1.06)	0.07	0.53 (−0.03, 1.08)	0.06
Multiple pituitary hormone deficits	−0.65 (−1.25, −0.06)	0.03	−0.14 (−1.05, 0.77)	0.76	−0.12 (−1.11, 0.87)	0.81		
Mental health disorders	−0.12 (−0.50, 0.26)	0.54			−0.04 (−0.60, 0.53)	0.89		
Medications								
Metformin	0.12 (−0.19, 0.43)	0.44			−0.24 (−0.67, 0.19)	0.27		
Melatonin	0.25 (−0.28, 0.79)	0.35			0.67 (−0.08, 1.41)	0.08	0.25 (−0.50, 1.01)	0.51
Methylphenidate	0.42 (−0.27, 1.11)	0.23			−0.63 (−1.60, 0.35)	0.20		
Levothyroxine	−0.58 (−1.12, −0.02)	0.04	−0.27 (−1.08, 0.54)	0.51	−0.25 (−1.13, 0.64)	0.58		
Hydrocortisone	−0.66 (−1.25, −0.06)	0.03	Omitted due to collinearity		−0.13 (−1.12, 0.85)	0.79		
Growth hormone	−0.44 (−1.14, 0.26)	0.22			−0.01 (−1.14, 1.13)	0.99		
Insulin	−0.01 (−0.63, 0.62)	0.99			0.06 (−0.92, 1.05)	0.90		

^a^ The units of the beta coefficient are the SD of the difference between BMI *z*‐score at baseline and the follow‐up measure for the cohort. As such, a negative value suggests the predictor is associated with a greater reduction in BMI z‐score, whereas a positive value suggests this predictor is associated with less reduction in BMI *z*‐score.

^b^ Index of multiple deprivation is a measure of social deprivation based on geographic postcode of home address (https://imd‐by‐postcode.opendatacommunities.org/imd/2019); a score of 1 represents the most deprived decile and 10 the most affluent decile.

^c^ Dysglycaemia includes patients with a diagnosis of any one of type 1 diabetes mellitus, type 2 diabetes mellitus, prediabetes and/or insulin resistance.

^d^ Includes both confirmed and suspected diagnoses where assessment is awaited.

## 4 Discussion

We have characterized the largest reported real‐world cohort of adolescents living with obesity treated with semaglutide. The average 12‐month weight loss of 10 kg is lower than reported in the STEP‐TEENS trial [1], but remains clinically meaningful. Real‐world studies of GLP‐1RA in adults have also reported lower drug effectiveness compared with trials [5]. Our cohort was more diverse than the STEP‐TEENS trial; 20% reported mental health diagnoses and would have been excluded from that trial. Nearly half of our patients had confirmed or suspected autism. Obesity prevalence is high in autistic children [6], but this substantial proportion might reflect increased referrals for those already under medical surveillance and behavioural barriers limiting the success of lifestyle interventions [6], leading to use of pharmacotherapy.

Dose differences could account for the lower weight loss in our cohort. In STEP‐TEENS, all participants followed a standard 16‐week titration schedule up to 2.4 mg/week. In contrast, few of our patients were taking 2.4 mg/week, reflecting initial 1 mg/week licensing restrictions and clinical decisions driven by treatment response or side effects. Paradoxically, we observed greater weight loss in patients prescribed lower doses. This likely reflects clinicians holding lower doses for patients successfully losing weight, but may also be confounded by adherence, where poor adherence led to less weight loss with responsive dose escalation. Real‐world prescribing data in adults also suggests submaximal prescribing is common [5, 7]. While achieved weight loss may have been greater with higher dosing, pharmacokinetic modelling suggests that 1.7 mg/week can achieve clinically meaningful BMI reductions compared to placebo, albeit less than observed with 2.4 mg/week [8].

We sought to establish patient characteristics associated with treatment response as this could facilitate patient selection for treatment and support medication counselling. We chose ΔBMIz as the outcome measure as semaglutide use in children may slow weight gain (rather than weight loss) during linear growth, with resulting BMIz reduction. However, there are limitations of this metric: older adolescents can achieve a small reduction in BMIz with age even when BMI remains unchanged and the mathematical compression of BMIz at the extremes of distribution can minimize true differences between children with high BMI.

Female sex was associated with a greater 12‐month reduction in BMIz, mirroring trends observed in adults [9, 10]. Patients with T2DM had a smaller BMIz reduction at 6 months, although this did not persist at 12 months in a smaller cohort. Lower achieved weight loss with semaglutide in those with T2DM has also been reported in adults [10, 11]. Importantly, confirmed or suspected autism was not associated with treatment outcome. Self/parent‐reported emotional eating was associated with attenuated BMIz reduction, suggesting these individuals may benefit from adjunctive therapies to maximize weight management. Emerging adult data indicate GLP‐1RA may benefit binge eating behaviours [12], but evidence in adolescents is needed.

This study has limitations inherent to the retrospective design. Treatment schedules were not standardized, current maximum licensed doses were often not used, and adherence data were unavailable. Follow‐up was variable due to non‐attendance and the COVID‐19 pandemic. While we adjusted for actual treatment duration, this approach assumes a linear weight loss over time, which may not reflect true clinical trajectories. However, sensitivity analyses using unadjusted ΔBMIz identified the same associated factors. Anthropometric data were frequently missing for patients who discontinued treatment (Figure S1) and our completers‐only analysis may introduce attrition bias towards a greater treatment effect. Lifestyle advice, including diet and physical activity, was neither standardized nor systematically recorded, and causal attribution of the weight changes to semaglutide cannot be made as lifestyle changes may have contributed. Hyperphagia and emotional eating patterns were not formally assessed; this should be considered in future work. We did not analyse weight trajectories before starting semaglutide. Given adolescents with obesity typically have ongoing weight gain (as seen in the placebo group of STEP‐TEENS [1]), slowing the rate of gain would be a relevant outcome but not detectable in our analysis. Pubertal staging examinations were also not available; future studies should consider whether weight change differs by stage of puberty.

In conclusion, we observed a clinically important weight and BMIz change during 12 months of semaglutide treatment in adolescents, albeit less than reported in the only intervention trial in this age group. Patient factors associated with BMIz change during treatment were not consistently identified, although our findings suggest that adolescents with T2DM and emotional eating may achieve less reduction in BMIz, whereas females achieved greater loss. Further exploration of patient factors associated with response is required in both trial and observational settings.

## Funding

The authors have nothing to report.

## Conflicts of Interest

The authors declare no conflicts of interest.

## Supporting information


**Table S1:** Baseline demographics for all patients initiated on semaglutide, and those with weight measurements at 6 and 12 months.
**Table S2:** Medical diagnoses in the cohort.
**Table S3:** Weight and BMI changes after 6 and 12 months of semaglutide treatment by prescribed dose of semaglutide.
**Figure S1:** Data availability and loss to follow‐up in patients who started semaglutide treatment between June 2020 and September 2025.
**Figure S2:** Weight and BMI *z*‐score change after 6 (blue bars) and 12 (orange bars) of semaglutide treatment.

## Data Availability

The data that support the findings of this study are available on request from the corresponding author. The data are not publicly available due to privacy or ethical restrictions.
