# Supplementary material for: Semaglutide in Adolescents Living With Obesity: A Real‐World Data Study Exploring Predictors of Treatment Response
Source: Diabetes Obes Metab. 2026 Jun 3;28(8):7616–21. doi: 10.1111/dom.70926 (PMC13341411; doi:10.1111/dom.70926)

**Semaglutide in adolescents living with obesity: a real-world data study exploring predictors of treatment response**

**SUPPLEMENTARY APPENDIX**

Valeria Cimador^1,2^, Sophie Robertson^3^, Christine Desmond^1^, Jessica Dodgson^3^, Elizabeth van Boxel^1^, Nikki L Davis^1*^, Rebecca J Moon^1,4,5*^

^1^Regional Centre for Paediatric Endocrinology, Southampton Children’s Hospital, University Hospital Southampton NHS Foundation Trust, Southampton, UK

^2^Residency School of Pediatrics, University of Bologna, Bologna, Italy

^3^Department of Paediatrics, Portsmouth Hospitals University NHS Trust, Portsmouth, UK

^4^MRC Lifecourse Epidemiology Centre, University of Southampton, Southampton, UK

^5^NIHR Southampton Biomedical Research Centre, University of Southampton and University Hospital Southampton NHS Foundation Trust, Southampton UK

*NLD and RJM are joint senior author

**Tables**

**Supplementary Table 1:** Baseline demographics for all patients initiated on semaglutide, and those with weight measurements at 6 and 12 months.

|  | **All semaglutide initiations (n=220)** | | **With weight data at 6 months of treatment (n=161)** | | **With weight data at 12 months of treatment (n=85)** | |
| --- | --- | --- | --- | --- | --- | --- |
|  | n |  | n |  | n |  |
| Male sex, n(%) | 220 | 101 (45.9) | 161 | 74 (46.0) | 85 | 37 (43.5) |
| Age (years), median (IQR) | 220 | 15.1 (13.6, 16.7) | 161 | 15.1 (13.6, 16.7) | 85 | 14.5 (12.8, 15.8) |
| Weight (kg), mean (SD) | 217 | 113.0 (27.6) | 161 | 113.5 (28.3) | 85 | 113.0 (28.8) |
| Weight z-score, mean (SD) | 217 | 3.90 (1.02) | 161 | 3.92 (1.03) | 85 | 4.02 (0.99) |
| BMI (kg/m2), median (IQR) | 217 | 40.0 (7.3) | 161 | 40.3 (7.4) | 85 | 40.4 (7.7) |
| BMI z-score, mean (SD) | 217 | 3.55 (0.58) | 161 | 3.57 (0.57) | 85 | 3.61 (0.57) |
| IMD decile, n (%)  1  2  3  4  5  6  7  8  9  10 | 217 | 33 (15.2)  30 (13.8)  24 (11.1)  18 (8.3)  13 (6.0)  25 (11.5)  21 (9.7)  18 (8.3)  18 (8.3)  17 (7.8) | 159 | 23 (14.5)  24 (15.1)  18 (11.3)  14 (8.8)  8 (5.0)  19 (12.0)  18 (11.3)  14 (8.8)  10 (6.3)  11 (6.9) | 83 | 13 (15.7)  12 (14.5)  10 (12.1)  7 (8.4)  2 (2.4)  15 (18.1)  5 (6.0)  11 (13.3)  3 (3.6)  5 (6.0) |
| **Comorbidities** |  |  |  |  |  |  |
| Dysglycaemia^†^, n (%)  Insulin resistance  Prediabetes  Type 2 diabetes  Type 1 diabetes | 217 | 186 (85.7)  118 (54.4)  41 (18.9)  21 (9.7)  6 (2.8) | 161 | 142 (88.8)  92 (57.5)  33 (20.6)  17 (10.6)  6 (3.7) | 84 | 77 (91.7)  53 (63.1)  20 (23.8)  4 (4.8)  3 (3.5) |
| Hypertension, n (%) | 213 | 23 (10.8) | 160 | 18 (11.3) | 85 | 10 (11.8) |
| Metabolic dysfunction associated steatotic liver disease, n (%) | 215 | 103 (47.9) | 161 | 78 (48.5) | 85 | 42 (49.4) |
| Obstructive sleep apnoea, n (%) | 213 | 37 (17.4) | 159 | 28 (17.6) | 84 | 13 (15.5) |
| Autistic spectrum disorder^‡^, n (%) | 215 | 96 (44.7) | 159 | 72 (45.3) | 84 | 36 (42.9) |
| Attention deficit hyperactivity disorder ^‡^, n (%) | 211 | 57 (27.0) | 155 | 41 (26.5) | 83 | 23 (27.7) |
| Mental health disorders, n (%) | 217 | 43 (19.8) | 160 | 33 (20.6) | 84 | 15 (17.9) |

^†^dysglycaemia is a patient with any of T1DM, T2DM, prediabetes or insulin resistance. ^‡^Includes confirmed or suspected diagnoses.

**Supplementary Table 2** Medical diagnoses in the cohort

|  | N |
| --- | --- |
| **Respiratory** |  |
| Asthma | 20 |
| Primary ciliary dyskinesia | 1 |
| **Cardiac** |  |
| Structural congenital cardiac condition | 2 |
| **Gastrointestinal** |  |
| Crohn’s disease | 1 |
| Coeliac disease | 1 |
| **Endocrinology** |  |
| Growth hormone deficiency | 16 |
| Adrenal insufficiency | 14 |
| Primary hypothyroidism | 2 |
| Secondary hypothyroidism | 13 |
| Hyperthyroidism | 1 |
| Arginine vasopressin deficiency (diabetes insipidus) | 6 |
| Precocious puberty | 2 |
| Delayed puberty | 4 |
| Type 1 diabetes | 6 |
| Type 2 diabetes | 21 |
| Familial partial lipodystrophy | 1 |
| **Renal** |  |
| Chronic kidney disease | 5 |
| Renal transplant recipient | 3 |
| **Neurology** |  |
| Epilepsy | 5 |
| Non-epileptic attack disorder | 1 |
| Migraines | 1 |
| Intracranial hypertension | 5 |
| Cerebral palsy | 2 |
| Other acquired brain injury | 1 |
| Learning difficulties | 11 |
| **Oncology** |  |
| Leukaemia | 3 |
| Brain tumour (including craniopharyngioma) | 5 |
| **Other** |  |
| Juvenile idiopathic arthritis | 1 |
| Juvenile dermatomyositis | 1 |
| Scoliosis | 2 |
| Autistic spectrum disorder (confirmed or suspected) | 96 |
| Attention deficit hyperactivity disorder (ADHD) (confirmed or suspected) | 57 |
| Mental health disorders | 43 |

**Supplementary table 3:** Weight and BMI changes after 6 and 12 months of semaglutide treatment by prescribed dose of semaglutide

|  | **After 6 months of treatment** | | | **After 12 months of treatment** | | |
| --- | --- | --- | --- | --- | --- | --- |
| **Dose** | **≤1mg/week** | **≥1mg/week** | **p** | **≤1mg/week** | **≥1mg/week** | **p** |
| **Weight (n)** | 138 | 21 |  | 60 | 24 |  |
| Weight loss (kg), mean (SD) | 6.5 (7.2) | 5.4 (6.7) | 0.52 | 10.6 (11.6) | 8.7 (11.6) | 0.49 |
| Weight loss relative to starting weight (%), median (IQR) | 5.1 (1.0, 8.8) | 4.2 (1.0, 7.1) | 0.54 | 9.8 (2.6,15.0) | 5.7 (-0.2, 13.9) | 0.27 |
| ≥5% weight loss, n (%) | 71 (51.5) | 10 (47.6) | 0.74 | 41 (68.3) | 14 (58.3) | 0.38 |
| ≥10% weight loss, n (%) | 30 (21.7) | 3 (14.3) | 0.43 | 29 (48.3) | 9 (37.5) | 0.37 |
| ≥15% weight loss, n (%) | 13 (9.4) | 0 (0.0) | 0.14 | 15 (25.0) | 5 (20.8) | 0.69 |
| **BMI (n)** | 137 | 21 |  | 59 | 23 |  |
| BMI reduction (kg/m^2^), mean (SD) | 2.7 (2.5) | 2.1 (2.0) | 0.30 | 4.6 (3.9) | 3.6 (3.4) | 0.33 |
| BMI reduction relative to starting BMI (%), mean (SD) | 8.5 (9.4) | 5.8 (9.0) | 0.23 | 11.3 (9.4) | 8.5 (7.5) | 0.20 |
| BMI z-score reduction, median (IQR) | 0.22 (0.11, 0.43) | 0.18 (0.05, 0.23) | 0.10 | 0.41 (0.19, 0.77) | 0.29 (0.08, 0.55) | 0.13 |

**Supplementary Figures**

**Supplementary Figure 1:** Data availability and loss to follow-up in patients who started semaglutide treatment between June 2020 and September 2025


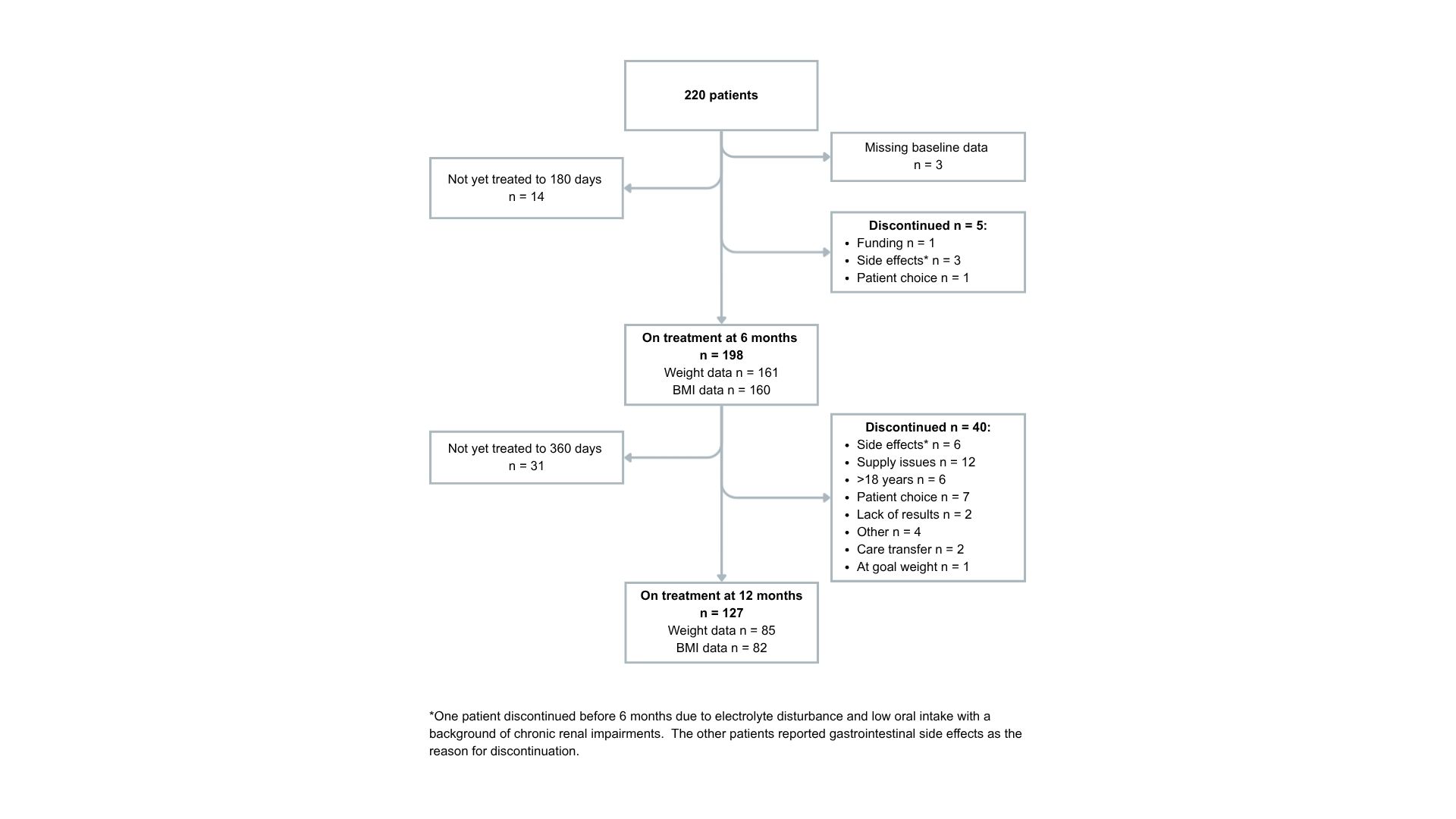


**Supplementary Figure 2:** Weight and BMI z-score change after 6 (blue bars) and 12 (orange bars) of semaglutide treatment.

Each bar represents an individual patient, with the data for 12 months (orange) overlying the data at 6 months (blue), where available. The green bars show weight and BMD z-score change for patients who did not have data available at 6 months.


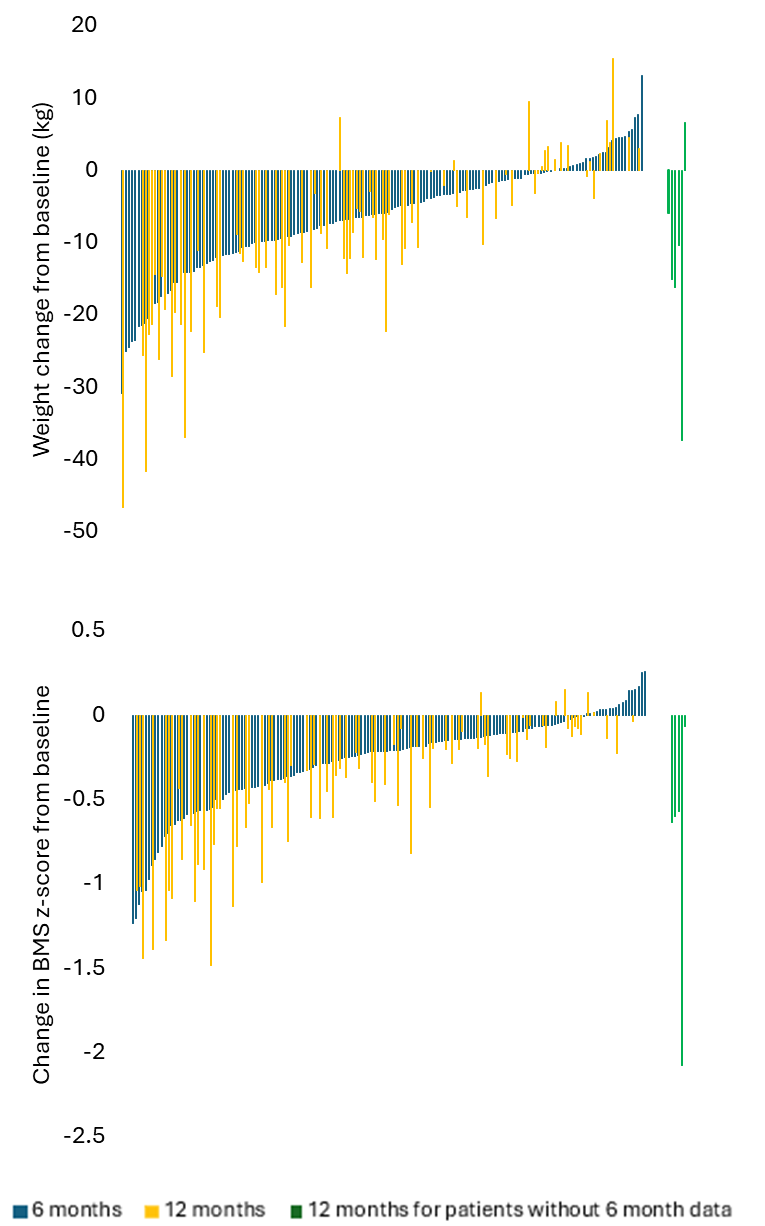

Supplement: Supplementary file 1 — Table S1: Baseline demographics for all patients initiated on semaglutide, and those with weight measurements at 6 and 12 months. Table S2: Medical diagnoses in the cohort. Table S3: Weight and BMI changes after 6 and 12 months of semaglutide treatment by prescribed dose of semaglutide. Figure S1: Data availability and loss to follow‐up in patients who started semaglutide treatment between June 2020 and September 2025. Figure S2: Weight and BMI z‐score change after 6 (blue bars) and 12 (orange bars) of semaglutide treatment. [file DOM-28-7616-s001.docx]
